# Spraying 6-BA could alleviate the harmful impacts of waterlogging on dry matter accumulation and grain yield of wheat

**DOI:** 10.7717/peerj.8193

**Published:** 2020-01-09

**Authors:** Xiaoyan Wang, Daoming Liu, Mingmei Wei, Jianguo Man

**Affiliations:** 1 Agronomy College, Yangtze University, Jingzhou, Hubei, China; 2 Key Laboratory of Crop Ecophysiology and Farming System in the Middle Reaches of the Yangtze River, College of Plant Science and Technology, Huazhong Agricultural University, Wuhan, Hubei, China

**Keywords:** Waterlogging, Grain yield, Wheat, Exogenous 6-benzylaminopurine

## Abstract

**Background:**

The middle and lower reaches of the Yangtze River plain produce the second highest amount of wheat in China; however, waterlogging is an important environmental factor that substantially affects the yield production of wheat (*Triticum aestivum* L.) in this region.

**Methods:**

In this study, seven treatments were implemented, including no waterlogging and exogenous 6-benzylaminopurine (6-BA) as a control (CK); waterlogging at booting (BW), anthesis (AW) and 15 days after anthesis (DAA, FW); and spraying 6-BA before waterlogging at booting (BW-6BA), anthesis (AW-6BA) and 15 DAA (FW-6BA), to determine the ability of 6-BA to alleviate the harmful impact of waterlogging on aboveground biomass production and grain yield. The widely cultivated wheat cultivar “Zhengmai 9023” was used.

**Results:**

The results showed that more than 190.0 mm of rainfall, which accounted for approximately 45.0% of the precipitation over the whole wheat growing season, was distributed after the booting stage (April and May). In all waterlogged treatments, the photosynthetic rate, aboveground biomass and grain yield decreased, but the differences between the CK and the FW treatment were not significant. The grain yield decreased by 18.38%, 41.79% and 5.67% in the BW, AW and FW treatments, respectively. Spraying 6-BA before waterlogging enhanced the root activities after anthesis and then decreased the malondialdehyde concentrations of the flag leaves and the third leaf, increased the photosynthetic rate of the flag leaves and enhanced aboveground biomass and grain yield. Among the increments between the treatments, the increments between the BW and BW-6BA treatments were the largest, but between the FW and FW-6BA were smallest. In comparison to the other waterlogging treatments, the grain yields from the FW and FW-6BA treatments were significantly higher because of the higher kernel numbers per spike. The results indicated that waterlogging after the booting stage restrained the dry matter production of winter wheat, but spraying 6-BA before waterlogging slowed the plant senescence rate and reduced grain yield loss.

## Introduction

Waterlogging is a severe worldwide challenge for crop production due to more frequent extreme climate events, such as continuous rain and storm (Yang et al., 2017; Wang, Liu & Jiang, 2017). It is indicated that 10–15 million ha of wheat are affected by waterlogging each year (Sayre et al., 1994) and this represents 15–20% of the 70 million ha sown to wheat each year (Setter & Waters, 2003). In China, waterlogging affects approximately 20–35% of agricultural soils (Jiang et al., 2008). Setter & Waters (2003) reported that large areas of waterlogging occur in the irrigated rice–wheat rotation systems including Pakistan, India, Nepal, Bangladesh and China. One major reason for this is that the soil preparation used for rice cultivation specifically results in subsoil compaction to optimize flooding conditions for rice (Samad et al., 2001), the other cause is the use of water containing high carbonate and bicarbonate concentrations which induces sodicity in these typically fine textured soils (Qureshi & Barrett-Lennard, 1998). With global climate change, waterlogging is predicted to increase in magnitude and frequency in the future, especially in mid- and high-latitude regions, where excessive wetness will increase with high precipitation events, such as in the Yangtze River Delta in China (Schumacher & Johnson, 2006; Jiang et al., 2008).

Waterlogging has a negative effect on wheat because gas exchange between the roots and the atmosphere is inhibited, oxygen concentration decreases rapidly in the root environment, while carbon dioxide and ethylene concentrations increase (Pampana, Masoni & Arduini, 2016). Waterlogging usually reduces wheat grain production by restricting root growth, photosynthesis and dry matter accumulation; promoting leaf senescence; and thus reducing kernel weight and grain yield (Brisson et al., 2002; Jiang et al., 2008; Hossain, Araki & Takahashi, 2011; Shao et al., 2013; Arduini et al., 2016; Pampana, Masoni & Arduini, 2016). Under waterlogged conditions, the death of roots due to O_2_ deficiency induces chlorophyl degradation and reduces photosynthesis, stomatal conductance and chlorophyl fluorescence (Zheng et al., 2009).

Winter cereal tolerance to waterlogging is related to factors such as: (i) the duration of the waterlogging event, (ii) the crop development stage in which waterlogging occurs and (iii) the sensitivity of the species or cultivar (De San Celedonio, Abeledo & Miralles, 2014; Arduini et al., 2016). Firstly, the causes of grain yield losses depend on the duration of the waterlogging are different, but mainly due to genotype and environment, generally the longer the waterlogging, the greater the grain yield decrease (Sharma & Swarup, 1988; Ghobadi & Ghobadi, 2010). Secondly, the responses of wheat to the development stage in which waterlogging occurs are also differences (Meyer & Barrs, 1988; Brisson et al., 2002; De San Celedonio, Abeledo & Miralles, 2014). Setter & Waters (2003) found that the tolerance to waterlogging during grain filling stage is greatest, following is at tillering stage and the worst is at seedling stage. Araki et al. (2012) demonstrated that waterlogging occurring after anthesis rather than waterlogging at the jointing stage had a more negative impact on yield. Arduini et al. (2016) and Pampana, Masoni & Arduini (2016) observed that there were no differences in waterlogging response at 3-leaf and 4-leaf stages. And they also reported that there were high genotypic differences for tolerance to waterlogging in wheat.

The middle and lower reaches of the Yangtze River plain (MLYR), where the study was conducted in this paper, is the second largest wheat production areas in China, accounting for more than 25.0% of the total wheat planting area in China. However, waterlogging often occurs after jointing of wheat (approximately 500–800 mm precipitation occurred from March to May), which significantly limit wheat grain production in this region (Zhao et al., 2007; Jiang et al., 2008). It has been reported that the grain yield per plant of wheat is the product of the number of spikes per plant, the number of spikelets per spike, the number of kernels per spikelet and the mean kernel weight (Arduini et al., 2016). Researchers found that, waterlogged during tillering stage of wheat, grain yield losses are mainly caused by a decrease in kernel number per spike (De San Celedonio, Abeledo & Miralles, 2014; Marti, Savin & Slafer, 2015; Arduini et al., 2016), or in kernel weight per plant (Ghobadi, Ghobadi & Zebarjadi, 2011), or by a combined reduction in the number of spike and kernel number per plant (Collaku & Harrison, 2002). To our knowledge, there were less researches were carried out to evaluate the effect of waterlogging after booting stage on wheat production in rice–wheat systems.

Researchers attempted to adopt some practices that could help crops coping with waterlogging. Zhan (2011) constructed horizontal, vertical and surrounding ditches in a wheat field and these ditches rapidly lowered the water table in the plow horizons after rainfall and then drained the rainfall out of the wheat field. Yang et al. (2017) used a new method of a ditch-buried rice straw return system to reduce the effects of waterlogging damage on wheat growth. In addition, other methods have also been implemented, such as applying N fertilizer (Li et al., 2006; Xie et al., 2003), or applying growth regulators (such as exogenous 6-benzylaminopurine (6-BA)) after waterlogging (Jiang et al., 2008; Ren et al., 2016). These measures could delay leaf senescence and increase chlorophyl content, improving the grain yield. However, it is usually difficult to apply nitrogen or 6-BA after waterlogging because of the extremely wet field.

In this study, waterlogging usually occurred after booting stage of wheat, we hypothesized that (1) the reduction the grain yield of wheat (*Triticum aestivum* L.) are likely due to the reduction the spikelet formation and kernel weight that caused by waterlogging, (2) the amount of reductions are related to the stages of waterlogging and (3) spraying 6-BA before waterlogging could alleviate the damage from waterlogging. Thus, we set seven treatments including three waterlogging stages, with or without spraying 6-BA before waterlogging, the treatment without waterlogging and 6-BA as a control. The aims are to investigated the impacts of waterlogging stages on wheat yield production and the alleviate effect of 6-BA at different stages.

## Materials and Methods

### Experimental site

The field experiment was imposed during the two cropping seasons of wheat from 2011 to 2013 at the Jingzhou experimental station of Yangtze University, Jingzhou, Hubei, China. This station is located in the center of the MLYR and its environment is typical and representative of the plain. The area has a subtropical monsoon climate and the accumulated sunshine, annual average temperature and total precipitation are approximately 2,000 h, 16.5 °C and 1,150 mm (Climate of Jingzhou, 2016), respectively. The soil is paddy-soil (Cooperative Research Group on Chinese Soil Taxonomy (CRGCST), 2001). The pH is 6.4, organic matter (10.5 g kg^−1^), total nitrogen (1.1 g kg^−1^), available phosphorus (45.4 mg kg^−1^) and available potassium (80.3 mg kg^−1^) in the topsoil of the experimental plots were measured using the potassium dichromate colorimetric method, the Kjeldahl method, the sodium bicarbonate method and the ammonium acetate method, respectively, details can be found in Lu (2000).

**Figure 1:**
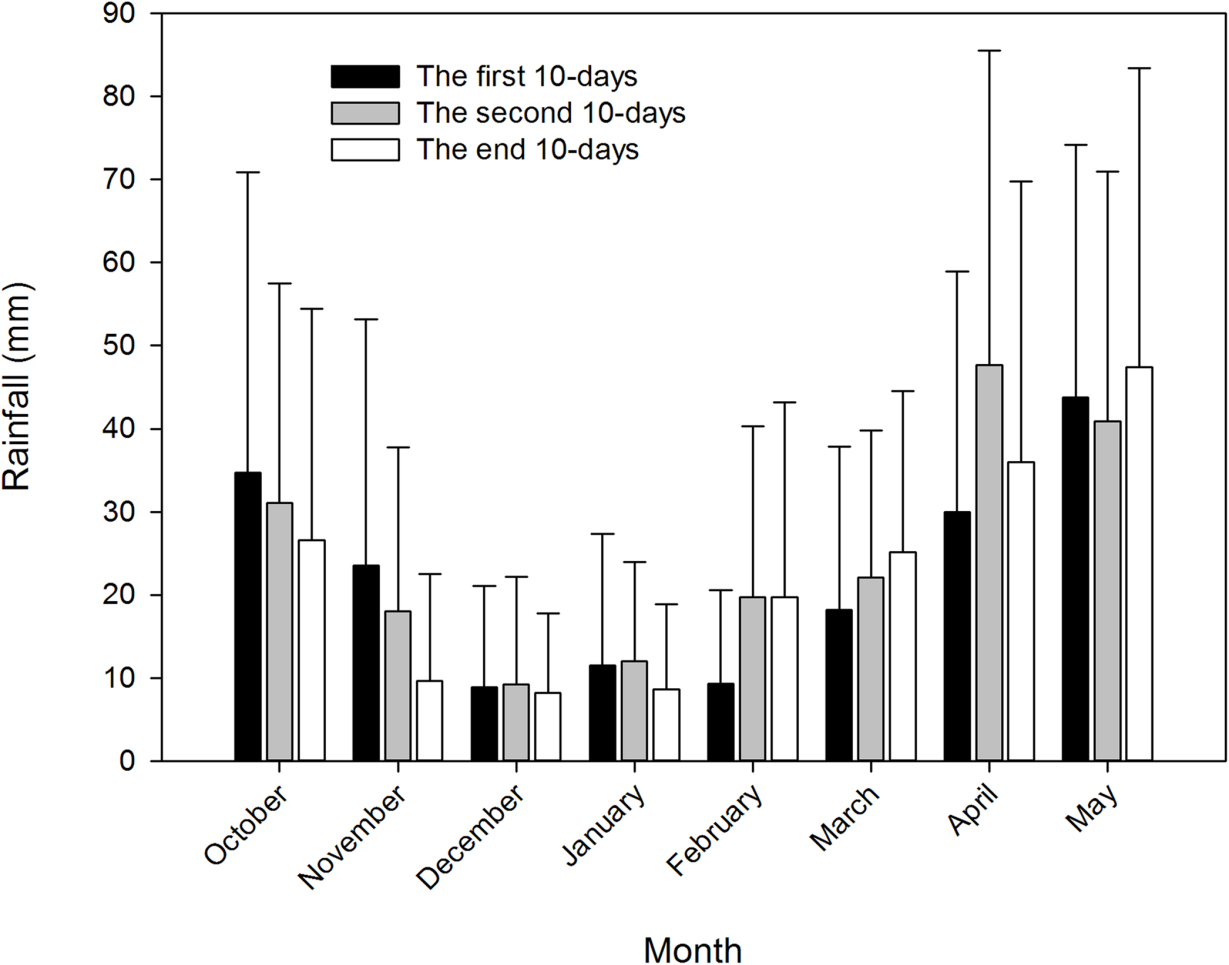
Rainfall in 10-days of each month during the whole wheat season at Jingzhou weather station from 1983 to 2012. Values of each month is the means from 1983 to 2012.

Average 10 days rainfall during winter wheat growing seasons from 1983 to 2012 is shown in Fig. 1. In this climate zone, wheat was sown after October 20 and harvested before May 30 in the next year. The 10 days rainfall decreased from the first 10 days of October to the end 10 days of December and then increased to 47.4 mm during the end 10 days of May. The average rainfall on the first, second and end 10 days of March was 18.2 mm, 22.1 mm and 25.1 mm, respectively; for the same days in April, the average rainfall was 29.9 mm, 47.7 mm and 36.0 mm, respectively; and in for the same days in May, the average rainfall was 43.8 mm, 40.9 mm and 47.4 mm, respectively.

### Experimental design

Seven treatments were arranged in a randomized complete block design with three times, the treatments are: without waterlogging and 6-BA as a control (CK), waterlogging at the booting stage (Zadoks 41, Table 1; Zadoks, Chang & Konzak, 1974) (BW), spraying 6-BA before waterlogging at the booting stage (BW-6BA), waterlogging at anthesis (Zadoks 60) (AW), spraying 6-BA before waterlogging at the anthesis stage (AW-6BA), waterlogging at 15 days after anthesis (DAA) (Zadoks 75) (FW) and spraying 6-BA before waterlogging at 15 DAA (FW-6BA). Thus, 21 plots were set for each wheat growing season. Each experimental plot was 2 × 6 m in size.

**Table 1:** Wheat major growth stages in the two growing seasons. Dates are mean values of treatments.

Stages	Zadoks scale	Growing season	
		2011–2012	2012–2013
Sowing	0	25 October 2011	8 November 2012
1st node detectable	31	2 March 2012	10 March 2013
Flag leaf sheath extending (early-booting stage)	41	25 March 2012	29 March 2013
Beginning of anthesis	60	8 April 2012	12 April 2013
Medium milk (15 DAA)	75	23 April 2012	27 April 2013
Late milk (25 DAA)	77	3 May 2012	7 May 2013
Maturity	92	10 May 2012	13 May 2013

Waterlogging plots were insulated with a plastic bezel, which was made of polyvinyl chloride. The plastic bezel was buried deeply, that is, 40 cm belowground and it extended 20 cm aboveground. For each plot, waterlogging was conducted through artificial means at booting, anthesis and 15 DAA, respectively, with 2 cm water layer above the ground and the water layer lasted for 7 days. At the end of each waterlogging period, water was drained out of each plot, after which they were maintained near to field capacity until the plants reached maturity. Control plots were watered near to field capacity throughout the growing season.

For the treatments with spraying 6-BA, 6-BA (supplied by China Medical Technology Co. Ltd., the concentration was 0.01 mmol) was sprayed 2 or 3 days before waterlogging.

### Crop management

The experimental variety is “Zhengmai 9023,” which has been widely planted in this area. The previous crop is rice. All plots were supplied with 180 kg N ha^−1^, 105 kg P_2_O_5_ ha^−1^ and 105 kg K_2_O ha^−1^. All P fertilizer (Superphosphate) and K fertilizer (Potassium chloride) and half the N fertilizer (Urea) were applied presowing by manure drill and the remaining N fertilizer (Urea) was top-dressed at the jointing stage by manual. Wheat seeds were sown by drill (width is 2.0 m) with space between two wheat rows was 0.2 m on 25 October 2011 and 8 November 2012, respectively. Seedlings were thinned out to a density of 210 plants m^−2^ at three-leaf stage in each plot every year.

Weed control was performed throughout the two crop cycles by hand hoeing. The occurrence of diseases was checked weekly throughout the growth cycles.

### Weather data

Weather data, including daily sunshine duration, average temperature and rainfall, were collected by the automatic meteorological station (CR800; Campbell Scientific Inc., Logan, UT, USA), which was located nearby the experiment field.

### Photosynthetic rate

Ten flag leaves from each experimental plot were selected. The photosynthetic rate (*P*n) of the flag leaves were determined using a CIRAS-2 portable photosynthesis system (PP-Systems, Hitchin, UK) under natural conditions with 380–390 mg kg^−1^ CO_2_ and 1,100–1,200 μmol m^−2^ s^−1^ photosynthetically active radiation and at 28–30 °C. Measurements were conducted between 9 and 12 am on days with full sunlight at booting, anthesis, 10 DAA and 28 DAA.

### Biochemical assays on leaves

In each plots, flag leaves and the third leaves (the leaf which counted from the top of wheat) were sampled with fifteen leaves at 25 DAA (Zadoks 77). Leaves from the plots were detached, immediately submerged in liquid nitrogen, and then stored at −80 °C until biochemical assays were performed.

Malondialdehyde (MDA) concentrations of the leaves were assayed according to Quan et al. (2004) and expressed as nmol g^−1^ fresh weight (FW).

### Root activity

Roots were sampled from each treatment (three replicates) were taken at booting, anthesis, 7 DAA and 28 DAA. Following to the description of Xue et al. (2003) and Zhang et al. (2009), we carefully removed the aboveground parts prior to root sampling and collected root samples from 0 cm to 20 cm soil layer. Two cores per plot were collected: one within the crop row and one midway between rows. The resultant mixture of roots and soil was then placed in a polythene bag and washed with tap water. According to Li et al. (2011b) the root activity was determined using the triphenyl tetrazolium chloride (TTC) method (Lindström & Nyström, 1987) and represented by the TTC reduction activity.

### Aboveground biomass

Plant samples were collected to determine aboveground biomass at maturity (Zadoks 92). On this occasion, 20 consecutive plants, making up one sample, were cut manually at ground level from each plot. These plants were separated into leaves, stems plus sheaths, spike axis plus glumes and grain at maturity. All samples were dried to a constant weight in a forced-draft oven at 70 °C and their dry weight was recorded.

### Yield and its components

Grain yield was determined from 4 m^2^ quadrat cuts from each plot and was reported on a 12.5% moisture content basis. All spikes from the sampled area (4 m^2^ quadrat) were counted to calculate spikes ha^−1^ for each plot. Forty plants were then randomly chosen from each plot and threshed by hand and the number of grains was counted to determine kernels per spike. Kernel weight was measured using 1,000 kernels. Harvest index is the ratio of grain yield to total aboveground biomass production at maturity.

### Statistical analysis

Twenty-one plots were arranged in a randomized complete block design, one-way analysis of variance (ANOVA) was determined using SPSS 13.0 software (SPSS Inc., Chicago, IL, USA). The significantly differences between treatments were tested by least significant difference method at probability level of 0.05.

A randomized complete block design was arranged in this experiment, thus, we use one-way standard analysis of variance method to determine the significantly differences between treatments. Although a spilt–split design was not adopt here, the differences between waterlogging stages, with and without spraying 6-BA at the same stages, could also be tested and highlighted.

## Results

### Climate factors during wheat growing seasons in 2011–2013

**Figure 2:**
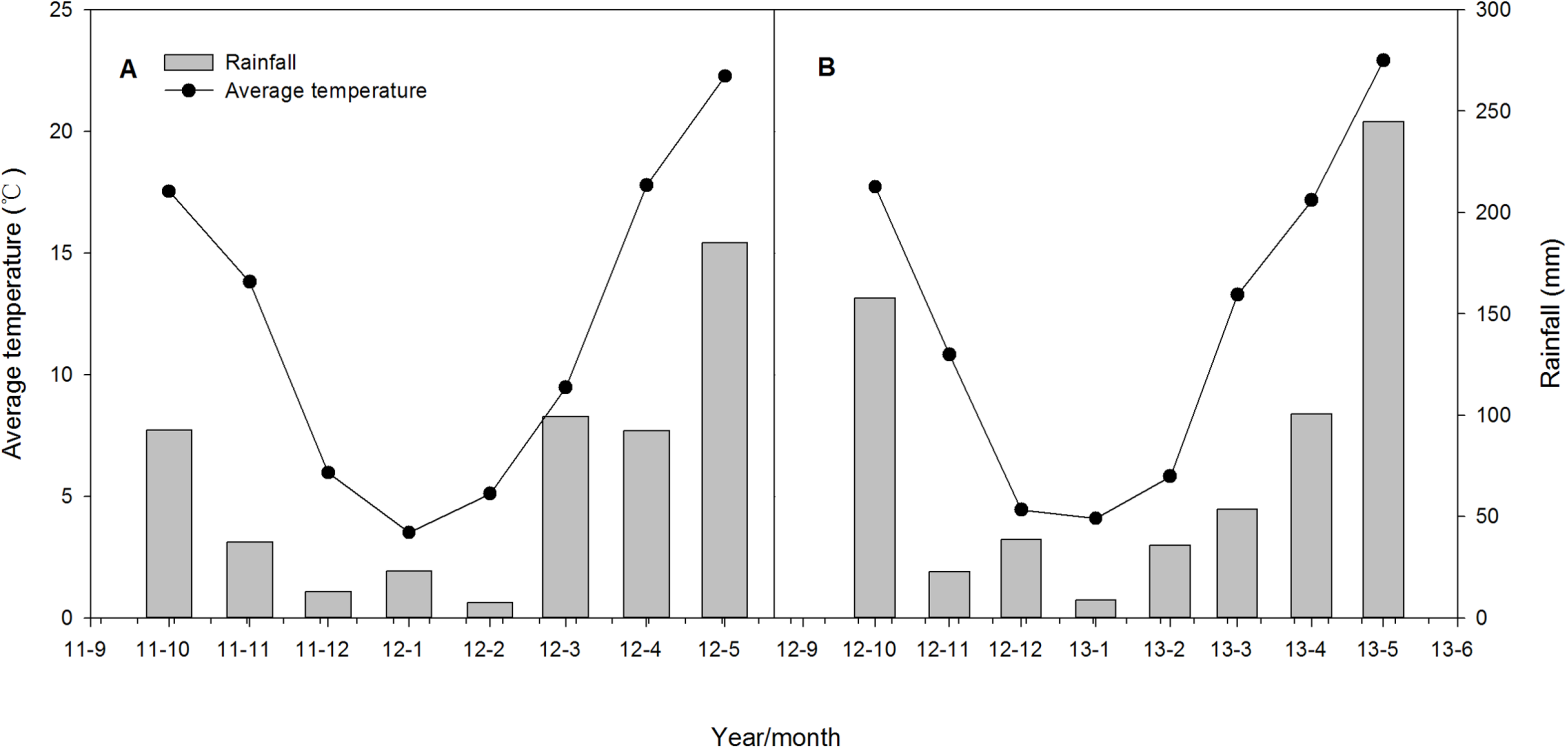
Monthly average temperature and total rainfall during wheat growing seasons at experimental station from 2011 to 2012 (A) and 2012 to 2013 (B) cycles.

The average temperature and rainfall of each month during the wheat growing seasons from 2011 to 2013 are shown in Fig. 2. The average temperature and total rainfall during wheat growing season in the 2011–2012 wheat season were 12.1 °C and 662.6 mm, respectively, which were 11.9 °C and 549.6 mm respectively in the 2012–2013 wheat season. The total rainfall was 376.4 and 398.6 mm from March and May in 2011–2012 to 2012–2013 growing season, respectively, higher rainfall in 2012–2013 because of the high precipitation levels on April 29 (57.3 mm) and May 7 (67.5 mm).

### Grain yield and yield components

The grain yield, kernel number per spike and 1,000 kernel weight of the two growing seasons were shown in Table 2. During both seasons, the CK treatment had the highest grain yield and kernel number per spike, the 1,000 kernel weight is a little lower than FW-6BA, but higher than the other treatments. For the waterlogging treatment, the grain yield, kernel number per spike and 1,000 kernel weight from FW treatments were significantly higher than those from BW and AW, BW treatment had higher grain yield and kernel number per spike than those from AW. For the treatments with 6-BA, the grain yield, kernel number per spike (except in 2011–2012 growing season) and 1,000 kernel weight from FW-6BA were the highest, those from AW-6BA were the lowest.

**Table 2:** Effect of different treatments on grain yield, kernel number per spike and 1,000-kernel weight.

Growth season	Treatment	Grain yield (t ha^−1^)	Kernel number per spike (Kernel spike^−1^)	1,000-Kernel weight (g)	Harvest index (%)
2011–2012	CK	5.44 ± 0.09	30.4 ± 0.03	44.9 ± 0.21	46.8 ± 1.4
	BW	4.42 ± 0.11	28.3 ± 0.08	39.2 ± 0.26	46.4 ± 1.3
	AW	3.27 ± 0.11	22.1 ± 0.12	40.7 ± 0.57	41.9 ± 1.4
	FW	5.12 ± 0.11	30.4 ± 0.05	44.7 ± 0.23	50.9 ± 2.4
	BW-6BA	4.92 ± 0.09	29.6 ± 0.05	42.8 ± 0.59	43.2 ± 1.7
	AW-6BA	3.56 ± 0.08	23.8 ± 0.18	42.5 ± 0.11	38.3 ± 2.5
	FW-6BA	5.20 ± 0.07	29.0 ± 0.12	45.0 ± 0.32	45.2 ± 1.5
	Mean	4.56	27.7	42.8	44.7
2012–2013	CK	5.16 ± 0.14	30.5 ± 0.35	41.3 ± 0.78	49.8 ± 2.6
	BW	4.21 ± 0.12	27.4 ± 0.19	38.2 ± 0.87	46.7 ± 1.4
	AW	2.90 ± 0.13	19.2 ± 0.13	39.5 ± 0.70	36.7 ± 1.6
	FW	4.87 ± 0.16	29.4 ± 0.05	40.0 ± 0.54	51.6 ± 0.5
	BW-6BA	4.59 ± 0.15	27.9 ± 0.18	39.9 ± 0.29	44.4 ± 1.0
	AW-6BA	3.04 ± 0.15	21.8 ± 0.15	41.1 ± 0.23	33.5 ± 1.1
	FW-6BA	4.95 ± 0.14	29.3 ± 0.25	42.0 ± 1.19	50.4 ± 0.6
	Mean	4.25	26.5	40.3	44.7

**Note:**

Values are mean ± standard deviation.

Spraying 6-BA before waterlogging could improve grain yield by increasing kernel number or kernel weight, but the increment related to waterlogging stage. The grain yield from BW-6BA, AW-6BA and FW-6BA were higher by 11.2%, 8.7% and 1.6% than that from BW, AW and FW treatments, respectively, in 2012–2013 growing season and higher by 8.8%, 5.1% and 1.6%, respectively, in 2012–2013 growing season.

### Aboveground biomass at maturity

**Figure 3:**
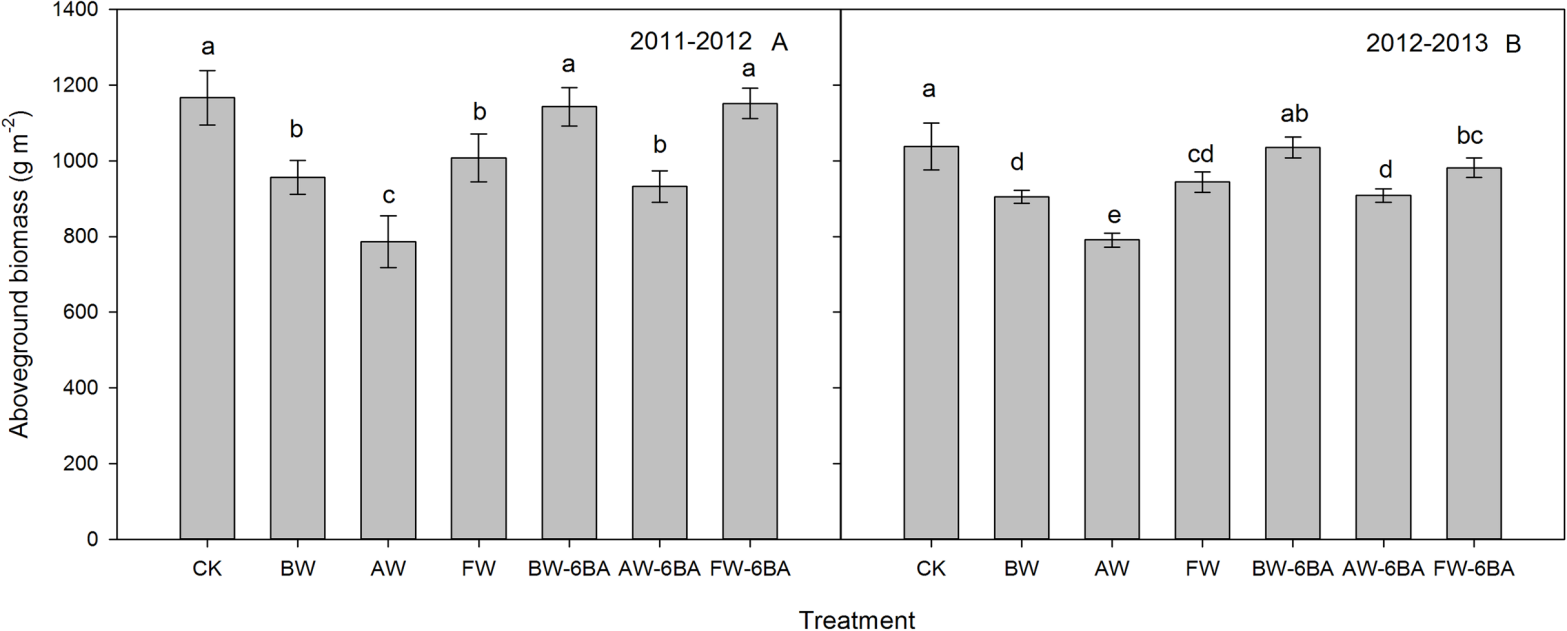
The effect of different treatments on the aboveground biomass production at maturity of 2011 to 2012 (A) and 2012 to 2013 (B) cycles.

The aboveground biomass of each treatment at maturity is shown in Fig. 3. The differences between treatments in the 2012–2013 growing season were similar to those in 2011–2012. In the 2011–2012 growing season, the CK had the highest biomass, at 11.66 t ha^−1^ and this amount of biomass had no differences with BW-6BA and FW-6BA treatments. For the waterlogging treatment, the grain yields, kernel number per spike and 1000 kernel weight from the FW were significantly higher than those from BW and AW. For the treatments with 6-BA, the biomass from BW-6BA and FW-6BA treatments were similar, but they were significantly higher than that from AW-6BA. Compared with BW, AW and FW, aboveground biomass from BW-6BA, AW-6BA and FW-6BA treatments were increased by 19.5%, 18.6% and 14.3%, respectively. The lowest amount of biomass occurred with the AW treatment at only 7.86 t ha^−1^.

### Photosynthetic rates of flag leaves

**Figure 4:**
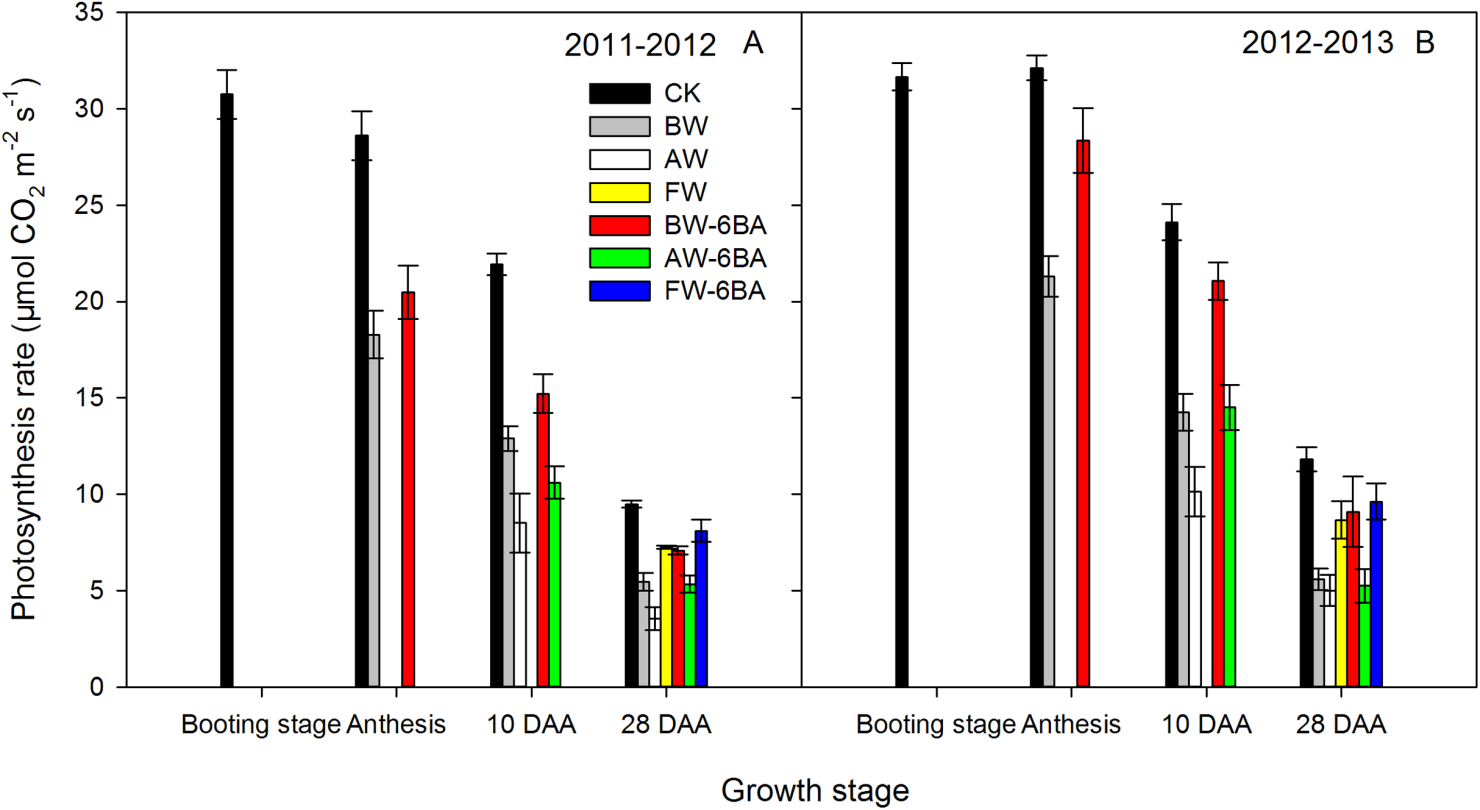
The effect of different treatments on photosynthetic rate of flag leaf after booting stage of 2011–2012 (A) and 2012–2013 (B) cycles.

The photosynthetic rates (*P*ns) of the flag leaves were measured at booting, anthesis, 10 DAA and 28 DAA in the two wheat growing seasons (Fig. 4). In both growing seasons, the *P*ns of the flag leaves from the booting stage to 28 DAA were higher in the CK treatment than in the other treatments. From anthesis to 28 DAA, the treatments with 6-BA had higher *P*ns than the treatments without 6-BA at the same stage and differences were significant after anthesis. At 7 DAA, the *P*ns of flag leaves were significantly higher in the BW and BW-6BA treatments than in the AW and AW-6BA treatments. At 28 DAA, the highest *P*n was obtained in the flag leaves from the FW-6BA treatment, followed by those from the FW treatment and then followed by those from the BW-6BA treatment. The values from the BW and AW-6BA treatments were not different but were significantly higher than those from the AW treatments.

### Malondialdehyde content of flag leaf and the third leaf

**Figure 5:**
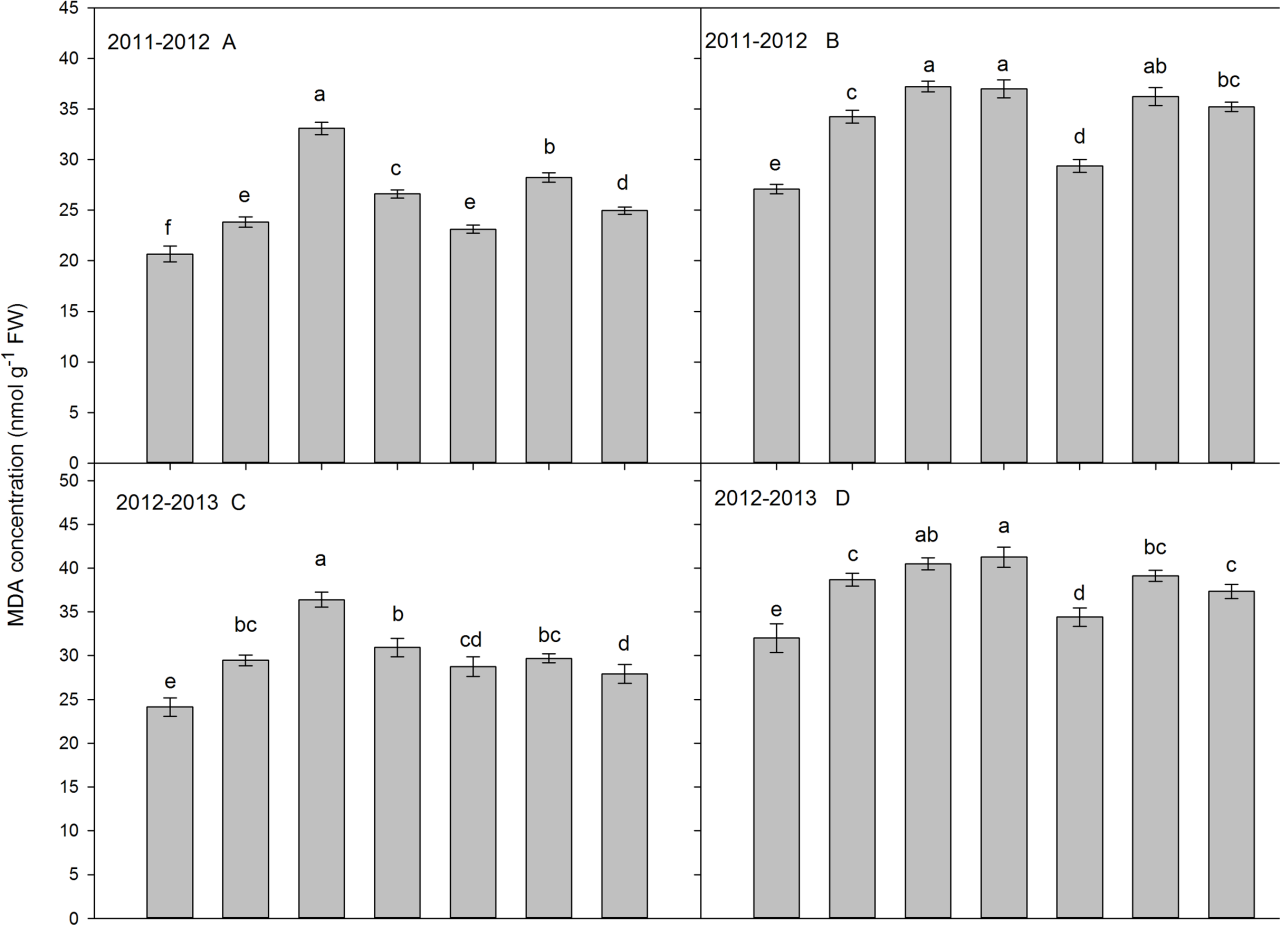
The effect of different treatments on MDA content in flag leaf (A and C) and the third leaf (B and D) at 25 DAA (2011–2012 and 2012–2013).

The MDA content of the flag leaves and the third leaf was measured at 25 DAA (Fig. 5). In both seasons, the MDA content either in the flag leaves or the third leaf was higher in the waterlogging treatment than in the treatments spraying 6-BA before waterlogging. The AW treatment had the highest MDA concentration in flag leaf and the third leaf, both in the 2011–2012 and 2012–2013 growing seasons. For the flag leaf, the order of differences in MDA content among treatments were AW-6BA > FW > FW-6BA > BW > BW-6BA > CK in 2011–2012 growing season. The differences were not significant between AW-6BA, FW, FW-6BA and BW treatments, but higher than that in BW-6BA and CK treatments. For the third leaf, the order of differences in MDA content among treatments were FW > AW-6BA > FW-6BA > BW > BW-6BA > CK in 2011–2012 growing season and FW > AW-6BA > BW > FW-6BA > BW-6BA > CK in 2012–2013 growing season. The MDA content in leaves from BW-6BA to CK treatments were significantly lower than the other treatments.

### Root activities after booting stage

**Figure 6:**
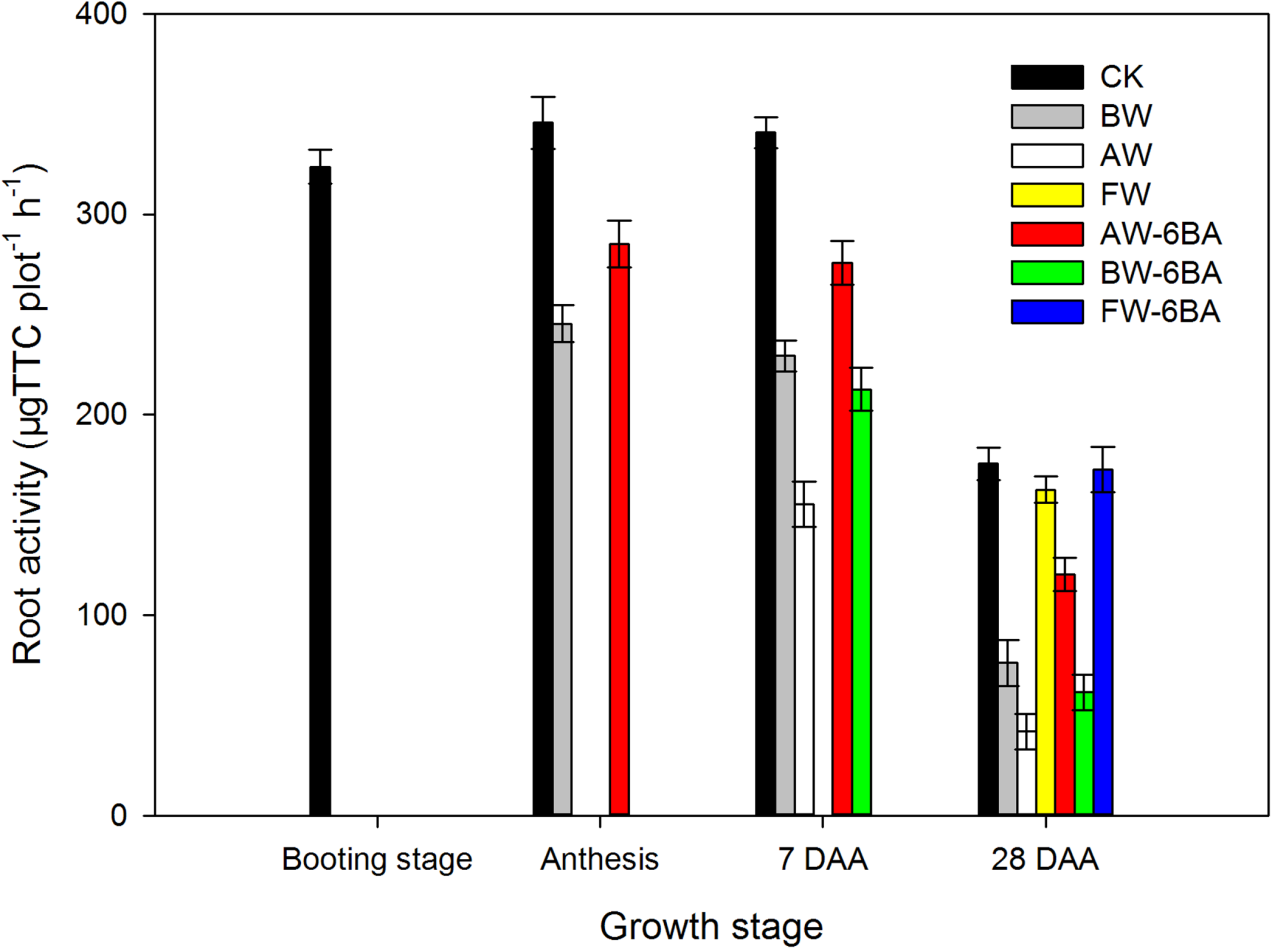
The effect of different treatments on the root activities at 0–20 cm soil layers after booting stage.

Root activities on the top 20 cm soil layers at booting, anthesis, 7 DAA and 28 DAA were measured (Fig. 6). At anthesis stage, the CK treatment had the highest root activity, followed by BW-6BA and the lowest was detected from BW. At 7 DAA, the activity of root from CK treatment was the highest, the treatment BW had higher root activity than AW, BW-6BA was higher than AW-6BA and higher root activities were detected from the treatments with spraying 6-BA than those treatment without 6-BA at same stage. At 28 DAA, the order of differences in root activities among treatments were CK > FW-6BA > FW > BW-6BA > BW > AW-6BA > AW.

## Discussion

### Relationship between waterlogging and grain yield components

More than 10% of the global land area is affected by waterlogging, which is one of the major abiotic factors limiting wheat production, particularly in the rice–wheat rotation regions of South and Southeast Asia, such as the MLYR of China (Arguello et al., 2016; Wu et al., 2018). In this experiment, the early-booting stage (Zadoks 41) of wheat occurred at 25 March 2012 and 29 March 2013 (Table 1), up to 376.4 mm of rainfall was accumulated from March to May both in 2012–2013 years (Fig. 2), therefore, excessive rainfall will happen after booting, which are likely increase the number of infertile florets (Kirby, 1990; Pampana, Masoni & Arduini, 2016), affect pollen fertilization and grain filling, finally reducing the grain yield (Fischer, 1993; Peltonen-Sainio, Jauhiainen & Hannukkala, 2007).

We hypothesized that the reduction the grain yield of wheat are likely due to the reduction the spikelet formation and kernel weight that caused by waterlogging. Number of kernels per spike is associated with number of spikelets per spike and number of florets per spikelet (Arduini et al., 2016). Spikelet initiation starts with the emergence of the fourth leaf (Zadoks 13) and ends at the stage of 1st node detectable (Zadoks 31) (Baker & Gallagher, 1983; Kirby, 1990), while, number of fertile florets is defined between terminal spikelet stage (Zadoks 31) and anthesis (Zadoks 60) (Kirby, 1990; Sinclair & Jamieson, 2006). In this study, waterlogging applied at booting stage (Zadoks 41), anthesis (Zadoks 60) and 15 DAA (Zadoks 75), respectively. Compared to CK treatment which was without waterlogging, the waterlogging treatments showed a significant reduction in grain yield, which were likely due to waterlogging affected the number of fertile florets after booting and finally reduce the kernel number per spike.

The mean kernel weight, which is mainly determined after anthesis, however, it was also affected by waterlogging after booting through decreased dry matter translocation (Wu et al., 2018). In this paper, the aboveground biomass at maturity from the waterlogging treatments was significantly lower than those from the CK and the lowest biomass amount was obtained from the AW treatment. In addition, the harvest index also decreased significantly in the waterlogging treatments (Table 2), especially in the AW treatment, compared to that in the CK. Therefore, carbohydrate translocation is negatively affected by waterlogging (Hossain, Araki & Takahashi, 2011), and the hypothesis that the waterlogging affect grain yield is likely due to the reduction the spikelet formation and kernel weight was supported by this experiment.

### Tolerance to waterlogging of wheat at different growth stages

Wheat tolerance to waterlogging is related to factors such as the duration of the waterlogging event, the crop development stage in which waterlogging occurs, and the sensitivity of the species or variety (Arduini et al., 2016). The duration of the waterlogging in this study was 7 days and a widely planted variety Zhengmai9023 was used, thus, we hypothesized that the development stages of wheat when waterlogging occurs are the mainly factors to waterlogging tolerance.

In common wheat, Arduini et al. (2016) found no differences in waterlogging response at 3-leaf and 4-leaf stages and well discussed why and which yield components were affected. Setter & Waters (2003) reported that in barley plants, the order of intolerance to waterlogging at different stages of development was grain filling > tillering stage > seedling stage. De San Celedonio, Abeledo & Miralles (2014) concluded that waterlogging occurring around anthesis reduced the yield of barley the most. In the present study, waterlogging was implemented after the booting stages. Among the treatments, the AW treatment resulted in the lowest grain yield, which was 39.9–43.8% lower than that in the CK, in both growing seasons. However, waterlogging during grain filling reduced yield to the smallest degree, which was only 5.6–5.8% lower than that in the CK, in both growing seasons. Therefore, the tolerance to waterlogging at grain filling stage is most, followed by booting stage, the worst is at anthesis of wheat, the result is in accordance with Araki et al. (2012) and De San Celedonio, Abeledo & Miralles (2014), who reported that waterlogging occurring near anthesis had the most negative impact on yield.

### Waterlogging promoted senescence of leaf and root

Waterlogging usually resulted in the reduction of stomatal conductance and CO_2_ in the leaves, promoting leaf senescence and finally reducing leaf photosynthesis (Jing et al., 2009; Li et al., 2011a; Wu et al., 2015). In this study, an improvement in MDA concentration of the flag leaves and the third leaf and a reduction in photosynthesis of the flag leaves were observed during waterlogging at the booting and anthesis stages, but these changes did not or only slightly occurred in FW treatment, compared with CK treatment. It indicates that waterlogging during the booting to anthesis growth phases promoted senescence of the leaves and reduced photosynthetic performance, while waterlogging during later phases had a smaller impact, which is consistent with Shao et al. (2013).

According to Haque, Oyanagi & Kawaguchi (2012), De San Celedonio, Abeledo & Miralles (2014), Arduini et al. (2016) and Sundgren et al. (2018), under waterlogging conditions, seminal roots of wheat are restricted or even die because of decreases in the oxygen concentration in the soil, which would limit water and nutrient uptake by roots, affect the balance between root and shoot growth and accelerate the shoot senescence or even lead to plant death. Setter & Waters (2003) concluded that much research has supported the benefits of adaptive traits for waterlogging including increases in aerenchyma and root porosity. However, not all of these are clearly shown to contribute to waterlogging tolerance of wheat and sometimes conflicting reports have occurred where different varieties or conditions have been used. This study only measured the root activities in the 0–20 cm soil layers and detected that the activities of roots in the AW treatment greatly declined, especially at the late grain-filling stages, however, the differences between the CK and the FW treatment were not significant. It indicated that waterlogging at the late developmental stages resulted in less damage to root and grain production. Unfortunately, we did not measure the dry-weight and aerenchyma in the root, thus waterlogging on the morphology and structure of root were unknown.

### Methods of improving the tolerance to waterlogging

Preventing senescence is an important approach to improve wheat yield under waterlogging conditions (Jiang et al., 2008; Li et al., 2011a; Ren et al., 2016; Gao et al., 2017; Yang et al., 2017). A ditch-buried rice-straw return method was designed by Yang et al. (2017), which was used to reduce the effects of waterlogging damage on wheat growth and maintain or increase wheat yields in rice-wheat rotation systems. Spraying exogenous hormones and N/P/K fertilizer after waterlogging could relieve the effects of waterlogging, that is, an 8.7% increase in chlorophyl content in flag leaves and a 61.4% increase in grain yield (Yang & Zhu, 2015). Spraying 6-BA after waterlogging for 6 days at the third leaf stage of maize could effectively improve grain-filling characteristics and photosynthesis of waterlogged summer maize, resulting in a significant increase in grain yield compared to those in water logging treatments (Ren et al., 2016). In this study, exogenous 6-BA was applied before each waterlogging treatment, which could significantly improve grain yield, increase kernel number per spike and 1,000 kernel weight, compared with the treatments without exogenous 6-BA at the same growing stage.

The rapidly decreasing trends in net photosynthesis and chlorophyl contents of flag leaves and in the amount of post-anthesis assimilated aboveground biomass were amended by spraying NaHSO_3_ and 6-BA after waterlogging (Xie et al., 2004). The root activities, flag leaf photosynthesis and aboveground biomass accumulation were higher and the MDA of the flag leaves and the third leaf were lower in the treatments with spraying 6-BA before waterlogging than in treatments without 6-BA during the same stage. These results are likely the reasons of high 1,000 kernel weight and grain yield in the treatments with spraying 6-BA before waterlogging.

After waterlogging, measures of response to waterlogging are usually difficult to carry out because of special field conditions. The results from this study showed that spraying 6-BA before a heavy rainfall is a feasible method to prevent or alleviate the negative impacts of waterlogging caused by excessive rainfall.

## Conclusion

This study show that waterlogging after booting stage of wheat decreased grain yield by reducing the kernel number per spike and lessening the kernel weight at maturity. The development stages in which waterlogging occurred had significantly effects on wheat production, anthesis stage was most susceptible to waterlogging, followed is at booting stage, the most insensitive stage is at mid-grain filling. This study also found that spraying 6-BA before waterlogging mitigated the decreasing trend of grain yield by slowing down the senescence rate of flag leaf and the third leaf, maintaining a high photosynthetic rate of flag leaf. These benefits are likely because of the high level of root activities during grain filling stages in the treatments with spraying 6-BA. The results of this study suggest that spraying 6-BA before waterlogging is a simple and effective approach to reduce the damage caused by waterlogging, especially from the booting to anthesis stages.

## Additional Information and Declarations

### Data Availability

The following information was supplied regarding data availability: Man, Jianguo (2019): Spraying 6-BA could alleviate the harmful impacts of waterlogging on dry matter accumulation and grain yield of wheat. figshare. Dataset. DOI 10.6084/m9.figshare.9800927.v1.
